# Synchrotron infrared nanospectroscopy in fourth-generation storage rings

**DOI:** 10.1107/S1600577524002364

**Published:** 2024-04-17

**Authors:** Thiago M. Santos, Sérgio Lordano, Rafael A. Mayer, Lucas Volpe, Gustavo M. Rodrigues, Bernd Meyer, Harry Westfahl Jr, Raul O. Freitas

**Affiliations:** a Brazilian Synchrotron Light Laboratory (LNLS), Brazilian Center for Research in Energy and Materials (CNPEM), 13083-970 Campinas, Sao Paulo, Brazil; b Gleb Wataghin Physics Institute, University of Campinas (Unicamp), 13083-859 Campinas, Sao Paulo, Brazil; Australian Synchrotron, Australia

**Keywords:** fourth-generation synchrotron, infrared nanospectroscopy, scattering scanning near-field microscopy, nano-FTIR, Imbuia at Sirius

## Abstract

Fourth-generation synchrotron storage rings represent a significant milestone in synchrotron technology, offering outstandingly bright and tightly focused X-ray beams for a wide range of scientific applications. However, due to their inherently tight magnetic lattices, these storage rings have posed critical challenges for accessing lower-energy radiation, such as infrared (IR) and THz. Here the first-ever IR beamline to be installed and to operate at a fourth-generation synchrotron storage ring is introduced. This work encompasses several notable advancements, including a thorough examination of the new IR source at Sirius, a detailed description of the radiation extraction scheme, and the successful validation of our optical concept through both measurements and simulations. This optimal optical setup has enabled us to achieve an exceptionally wide frequency range for our nanospectroscopy experiments. Through the utilization of synchrotron IR nanospectroscopy on biological and hard matter samples, the practicality and effectiveness of this beamline has been successfully demonstrated. The advantages of fourth-generation synchrotron IR sources, which can now operate with unparalleled stability as a result of the stringent requirements for producing low-emittance X-rays, are emphasized.

## Abstract 2

Infrared nanospectroscopy is demonstrated to be feasible in new-generation synchrotron storage rings.

## 1. Introduction

After decades of research, fourth-generation synchrotron storage rings have emerged as the most advanced form of synchrotron light sources (Einfeld *et al.*, 1995 ▸, 2014 ▸; Liu *et al.*, 2013 ▸; Revol, 2017 ▸). They possess the capability to generate intensely bright and tightly focused X-ray beams, which find applications across a wide spectrum of scientific research endeavors (Shin, 2021 ▸). This new generation of storage rings marks a significant leap in synchrotron accelerator technology (Liu *et al.*, 2014 ▸), delivering substantial improvements in beam stability, brilliance and transverse coherence lengths. A key feature of fourth-generation synchrotron storage rings is their remarkably reduced emittance (Johansson *et al.*, 2014 ▸), a property that enables focusing X-rays near their diffraction limit. This is essential for the development of multi-color X-ray nanoprobes (de Jonge *et al.*, 2014 ▸; Johansson *et al.*, 2014 ▸; Tolentino *et al.*, 2017 ▸). Furthermore, the high coherence of the X-ray beams empowers scattering, diffraction and spectroscopy stations to perform high-definition volume reconstruction, driving in a new era for X-ray imaging (Thibault *et al.*, 2014 ▸; Yabashi *et al.*, 2014 ▸).

To achieve these ultra-small emittances, these innovative accelerators rely on densely packed magnetic lattices, referred to as multi-bend-achromat (MBA) lattices (Johansson *et al.*, 2014 ▸; Borland *et al.*, 2014 ▸). The smooth bends of the MBA lattice connecting the straight sections ensure that electron bunches maintain their compactness under the influence of acceleration fields. Despite the tremendous progress in X-ray sources, creating low-energy stations at fourth-generation synchrotron storage rings is a non-trivial task. Longer wavelengths, such as visible, ultraviolet (UV) and infrared (IR), necessitate more physical space for the installation of large extraction ports due to their greater divergence (Moreno *et al.*, 2013 ▸). However, the tight configuration of the magnetic elements imposes severe spatial constraints, requiring the adaptation in the concept of previous IR extraction designs.

In retrospect, synchrotron IR programs are renowned for their multidisciplinary nature, versatility and cost-effectiveness, serving the synchrotron community by offering the power of chemical and vibrational contrast into both soft and hard matter analyses (Miller & Dumas, 2013 ▸; Dumas *et al.*, 2007 ▸; Holman *et al.*, 2010 ▸). Over the past decade, the combination of synchrotron IR and scattering scanning near-field optical microscopy (s-SNOM) (Zenhausern *et al.*, 1995 ▸; Kawata & Inouye, 1995 ▸; Knoll & Keilmann, 1999 ▸) has enabled broadband IR spectroscopy to achieve nanoscale resolutions, heralding a new era in IR analysis within large accelerators. Commonly referred to as SINS (Bechtel *et al.*, 2014 ▸) and derived from nano-FTIR (Huth *et al.*, 2012 ▸), synchrotron IR nanospectroscopy (Hermann *et al.*, 2013 ▸; Bechtel *et al.*, 2014 ▸; Freitas *et al.*, 2018 ▸) has played a pivotal role in addressing unresolved questions across various scientific domains, including biomedicine (Freitas *et al.*, 2021 ▸), nanophotonics (Barcelos *et al.*, 2015 ▸, 2020 ▸; Maia *et al.*, 2019 ▸), natural two-dimensional materials (Barcelos *et al.*, 2018 ▸; de Oliveira *et al.*, 2022 ▸, 2023 ▸), energy conversion materials (Szostak *et al.*, 2019 ▸), plant biology (Pereira *et al.*, 2018 ▸), modern batteries (Nepel *et al.*, 2021 ▸), catalysis (Wu *et al.*, 2017 ▸), electrochemistry (Macedo *et al.*, 2018 ▸), nano-drug delivery (Capeletti *et al.*, 2019 ▸), organics in liquid environments (Meireles *et al.*, 2019 ▸; Lu *et al.*, 2019 ▸) and bio-membranes (Berweger *et al.*, 2013 ▸). Despite the recent rapid evolution of synchrotron IR nanospectroscopy, which is now advancing towards even lower energies (Khatib *et al.*, 2018 ▸; Feres *et al.*, 2021 ▸; Barcelos *et al.*, 2021 ▸), this analytical approach is under the risk of discontinuation in novel fourth-generation machines due to the lattice requirements necessary to achieve extreme-low emittance X-rays.

In this work, we introduce the inaugural IR beamline operating in a fourth-generation synchrotron storage ring. Here we present the concept, technical specifics and initial quantitative results of the new synchrotron IR nanospectroscopy station (IMBUIA-nano) installed in the Sirius accelerator. We delve into the performance specifications of the light source, the concept of the radiation extraction and optical layout, providing in-depth details. The characterization of the beam has been verified and corroborated through numerical simulations. Following the coupling of the synchrotron beam with the s-SNOM, we conducted a series of nanospectroscopy experiments for a proof of performance on a polymer blend, protein nano-patches and a polar two-dimensional material. Our initial findings confirm a high sensitivity for the synchrotron s-SNOM experiment, even when operating with a reduced extraction port originally designed for visible-light diagnostics. Finally, we outline the future prospects for this station, including planned enlargement of the extraction port to enable far-IR and THz nano­spectroscopy.

## 2. Methods

### 2.1. IR synchrotron s-SNOM

The IR synchrotron beam was coupled to a commercial s-SNOM nanoscope (neaSCOPE, Attocube Systems AG), equipment that combines atomic force microscopy (AFM) and IR Fourier-transform infrared spectroscopy (FTIR) in the same sample environment for achieving IR nanospectroscopy. We used Pt-coated AFM tips (nano-FTIR, Attocube Systems AG) with ∼30 nm radius and mechanical resonance around 80 kHz. The optical signal collected by the detector was demodulated at the second harmonic of the tip’s natural frequency to suppress the far-field background. Wavenumber resolution was set at 10 cm^−1^. To remove the experimental influence (environment, detector sensitivity and tip radiating response) from the sample’s response, all data were normalized by a reference spectrum taken on a clean Au surface. Broadband reflectivity images were measured with the reference arm static at the interferogram center burst. Each spectrum presented later in Fig. 5 was Fourier processed from an interferogram measured with 2048 data points, 10 ms integration time per point and 40 averaging scans (∼14 min per spectrum). In Fig. 6, point-spectra were acquired with 1024 data points, 20 ms integration time per point and 30 averaging scans (∼10 min per spectrum). The linescan was set along a 1 µm line with 30 spectra, 1024 data points per interferogram, 20 ms integration time per point and 15 averaging scans per spectrum (∼2.5 h per linescan). In Fig. 7, interferograms were measured with 2048 data points, 10 ms integration time per point and 50 averaging scans (∼17 min per spectrum). Finally, a hexagonal boron nitride (hBN) point-spectrum, presented in Fig. 8, was acquired with 2048 data points, 10 ms integration time per point and 30 averaging scans (∼10 min per spectrum) whereas the linescan, set along a 3 µm line with 60 spectra, used 2048 data points per interferogram, 10 ms integration time per point and four averaging scans per spectrum (∼1.4 h per linescan). The UDR filters (Agilent Technologies, Inc) are coated germanium mid-IR lowpass filters which transmit below 3950 cm^−1^ (UDR4 filter) or 1975 cm^−1^ (UDR8 filter).

### 2.2. Sample preparation

A 100 nm-thick polymer blend of polystyrene/poly(vinyl acetate) on Si substrate (PS-PVAc/Si) was acquired from Park Systems Corporation. Bovine serum albumin (BSA) protein (A9418, Sigma-Aldrich) was diluted in water (0.05 mg l^−1^) and dispersed and dried on a clean Au surface producing nano­scale clusters. hBN 2D flakes were produced by double-sided tape mechanical exfoliation of a commercial hBN crystal (HQ Graphene).

### 2.3. Beam diagnostics

Direct diagnostics of the secondary source was achieved by using a CCD sensor (Manta G-125, Allied Vision Technologies GmbH) with 1024 × 1024 pixels equipped with a 980 nm bandpass filter (FB980-10, Thorlabs Inc.). Beam cross-section images at M5 and after collimation (downstream M6) were collected by projecting the beam on a ruled screen and by imaging it with a regular mobile camera.

### 2.4. Numerical simulations

**Figure 1:**
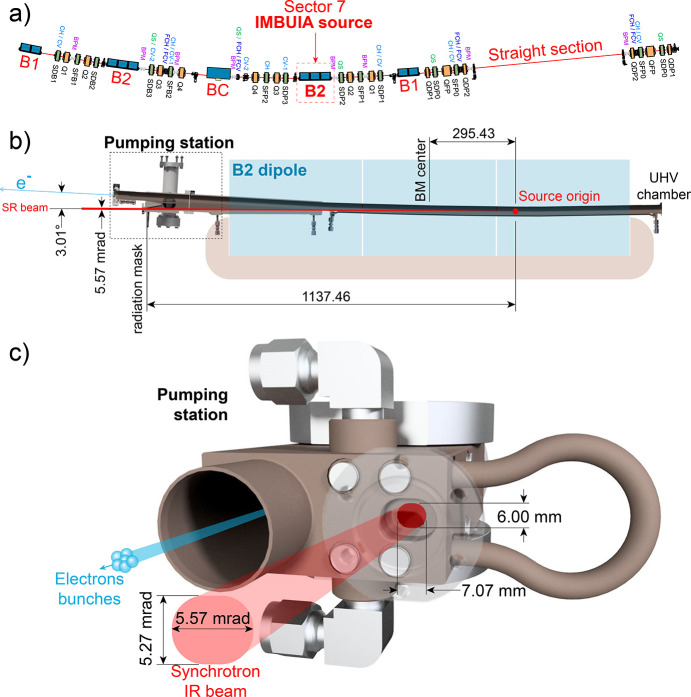
The IMBUIA lattice and IR extraction port. (*a*) Magnetic lattice of Sector 7 of the accelerator with indication of the bending magnet source (B2, 0.56 T). (*b*) Top-view section of the B2 dipole chamber highlighting the electrons’ orbit, synchrotron radiation extraction port (radiation mask) and source origin. (*c*) Pumping station after the dipole with radiation mask of 5.57 mrad × 5.27 mrad for synchrotron radiation extraction.

Numerical simulations were carried out using the open code *Synchrotron Radiation Workshop* (*SRW*), which accurately calculates the radiated emission from relativistic charged particles in arbitrary magnetic fields (Chubar & Elleaume, 1998 ▸). Since the beam is modeled in terms of scalar wavefields, *SRW* is suitable for simulating diffraction effects for beams of any coherence degree and polarization state, as they propagate through the beamline optics (Canestrari *et al.*, 2014 ▸). The source of the IMBUIA beamline is a low-field bending magnet (*B* = 0.56 T) with critical energy ε_c_ = 3.38 keV (B2 in the Sirius lattice). The dipole chamber extracts 5.6 mrad × 5.3 mrad (H × V) of the emission, from a specific region of the magnet defining the source point at 295 mm upstream of the dipole center (Fig. 1 ▸).

## 3. Infrared extraction and beamline optics

### 3.1. The source

Sirius is a 3 GeV storage ring currently operating at 100 mA (designed to reach 350 mA). Its five-bend-achromat (5BA) cell comprises two pairs of low-field (0.56 T) magnets, named B1 and B2, one super-bend (BC, peak field 3.2 T) and one straight section, as illustrated in Fig. 1 ▸(*a*). The IMBUIA beamline source is located in Sector 7 of the storage ring, inside the first B2 dipole downstream of the straight section. The bending magnet source is defined by an extraction port (originally designed for photon diagnostics in the visible range) centered at 3.01° from the electrons’ orbit [Fig. 1 ▸(*b*)] and longitudinally located at 295 mm upstream of the dipole center. A radiation mask of 7.07 mm × 6.00 mm located in the pumping station of the B2 dipole (1137 mm from the source) defines a radiation extraction of 5.57 mrad × 5.27 mrad [Fig. 1 ▸(*c*)].

**Figure 2:**
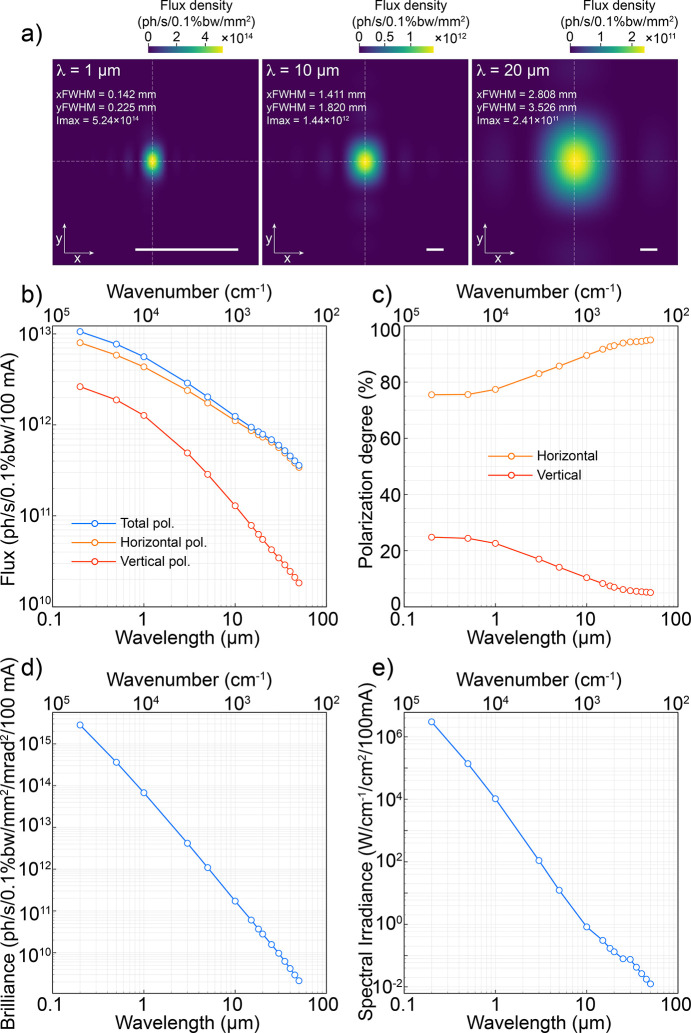
Source parameters and performance. (*a*) Numerical simulation of the source at wavelengths 1 µm, 10 µm and 20 µm. Scale bars represent 1 mm. (*b*)–(*e*) Calculated spectral flux, polarization degree, brilliance and irradiance in the visible–IR range from the extraction port described in Fig. 1 ▸. All results were simulated using *SRW*.

Taking into account the basic parameters of the magnetic fields in Sector 7 and the dimensions of the radiation mask, we numerically simulated the source using the open-source code *SRW*. In a first approach we simulated the source shape and flux density for 1 µm, 10 µm and 20 µm wavelengths [Fig. 2 ▸(*a*)]. Since the simulation becomes unstable as we get very close to the electron bunches, the simulations in Fig. 2 ▸(*a*) were obtained by applying ideal optics (1:1 magnification). The maximum collection angle was kept fixed following the port presented in Fig. 1 ▸. This allowed the reconstruction of the electric fields without solving the singularities near the charges. We found the size of the source to be directly proportional to the wavelength, therefore, it is a diffraction-limited point source in the infrared range. On the other hand, the maximum value for the flux density does not follow the same proportion law as it is influenced by the maximum collection angle of the port. To study this in depth, we calculated the spectral flux [Fig. 2 ▸(*b*)], polarization portions [Fig. 2 ▸(*c*)], spectral brilliance [Fig. 2 ▸(*d*)] and irradiance [Fig. 2 ▸(*e*)]. By observing the flux trend [Fig. 2 ▸(*b*)], it is clear that longer wavelengths are greatly affected by the small vertical aperture of the port, as the curve drops rapidly at wavelengths greater than 1 µm. Moreover, the extracted radiation is dominated by horizontal polarization, as the total polarization flux is just slightly above the horizontally polarized flux. This is confirmed by the polarization study in Fig. 2 ▸(*c*), where the horizontally polarized portion corresponds to more than 75% of the total. For longer wavelengths (>20 µm) the polarization becomes mainly horizontal (above 90%), confirming that a very small vertical portion of the far-IR/THz beam is passing through the extraction port.

Despite the reduced extraction port and high energy of the electrons (3 GeV) in comparison with our previous UVX machine (1.37 GeV), the smooth curve of the low-bend dipoles (reduced critical energy) produces competitive spectral brilliance and irradiance [Figs. 2 ▸(*d*) and 2(*e*)] in the mid-IR range. Those are comparable with standard large ports operated in low-energy storage rings, as achieved in our previous accelerator (Freitas *et al.*, 2018 ▸).

### 3.2. IR extraction elements

**Figure 3:**
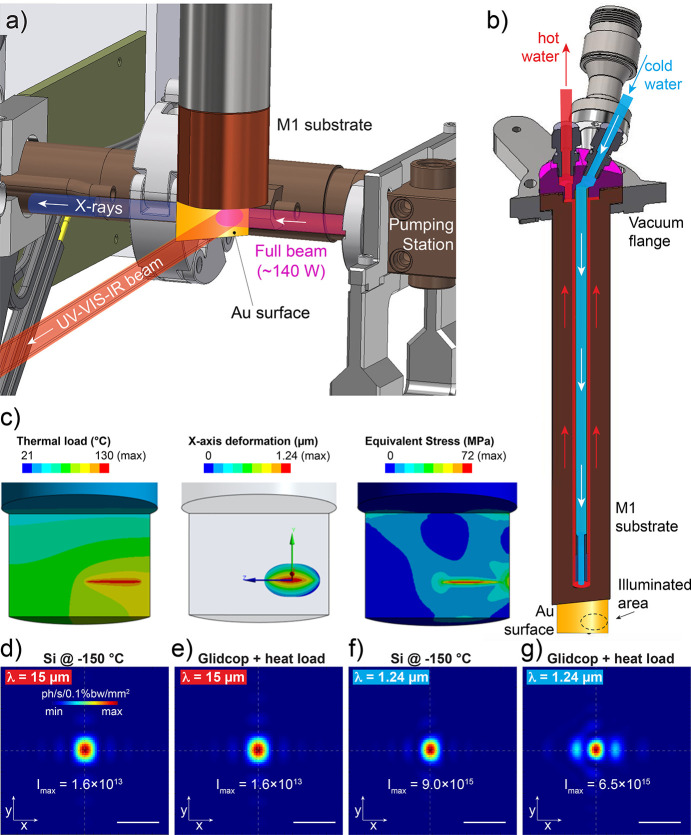
IR extraction mirror heat load. (*a*) A vertical-mounted flat mirror (M1) is illuminated by near 140 W of synchrotron radiation from the B2 bending magnetic. The Au-coated mirror surface reflects low-energy radiation (UV–VIS–IR) and transmits the high-energy portion (mostly X-rays) that is mainly absorbed by the Glidcop substrate. (*b*) Cross section of the M1 shaft highlighting the water-cooling flow path. (*c*) Numerical simulation of the thermal load, *X*-deformation and stress around the illuminated area of the mirror (left to right). (*d*–*g*) Optical simulation of the secondary source produced by the M1 flat mirror with a Si substrate at −150 °C (control case) and the Glidcop substrate (actual case with thermal bump) with the heat load and water-cooling system for λ = 15 µm (scale bars 1 mm) and λ = 1.24 µm (scale bars 100 µm).

For radiation collection, we designed a vertically supported flat mirror (M1) inserted from the top in front of the extraction port illustrated in Fig. 1 ▸(*c*). The full white beam from the bending magnet illuminates the Au-coated Glidcop flat surface that mostly absorbs the high-energy portion (X-rays) and reflects the lower-energy portion (UV, visible and IR), as illustrated in Fig. 3 ▸(*a*). As a safety requirement, the entire M1 shaft can move vertically in case the mirror needs to be removed from the beam path, which is the only degree of freedom of M1. Most of the heat load from the white beam is absorbed by M1, which corresponds to ∼140 W. Hence, a water-cooling system operates to drain this large amount of energy and to reduce M1 surface deformations. For that, a coaxial concept flows cold water (∼21°C, 150 ml min^−1^) towards the illuminated area guided by an inner pipe which returns warmer through the outer gap, as schemed in Fig. 3 ▸(*b*). The maximum temperature of the mirror surface is predicted to be about 130 °C for 350 mA (maximum storage ring current), as depicted in Fig. 3 ▸(*c*) (left panel). Given the total heat load and illumination area of the beam over M1, we calculated the deformation in the *X* direction (thermal bump quasi-normal to the surface) whose maximum is near 1.24 µm, as displayed in Fig. 3 ▸(*c*) (middle panel) sided by the equivalent stress that peaks at 72 MPa [Fig. 3 ▸(*c*), right panel].

To evaluate the impact of the thermal bump on the beam quality, we calculated the IR intensity (focal point) at the secondary source position (5.60 m from the primary source) considering M1 as an ideally flat liquid-nitrogen-cooled silicon substrate (Si at −150 °C) as a control case for 15 µm and 1.24 µm wavelengths [Figs. 3 ▸(*d*) and 3(*f*)], respectively. The same was done for the actual case, where M1 has a thermal bump due to the heat load, as presented in Figs. 3 ▸(*e*) and 3(*g*). For λ = 15 µm, no changes at all are noticed by comparing control and deformed M1. Yet, for λ = 1.24 µm, the focal point presents a slight aberration and a small decrease in the maximum intensity for the deformed case. Overall, the analysis confirms that the designed extraction optics does not introduce significant aberrations for wavelengths in the mid-IR range or longer wavelengths.

### 3.3. Optics

**Figure 4:**
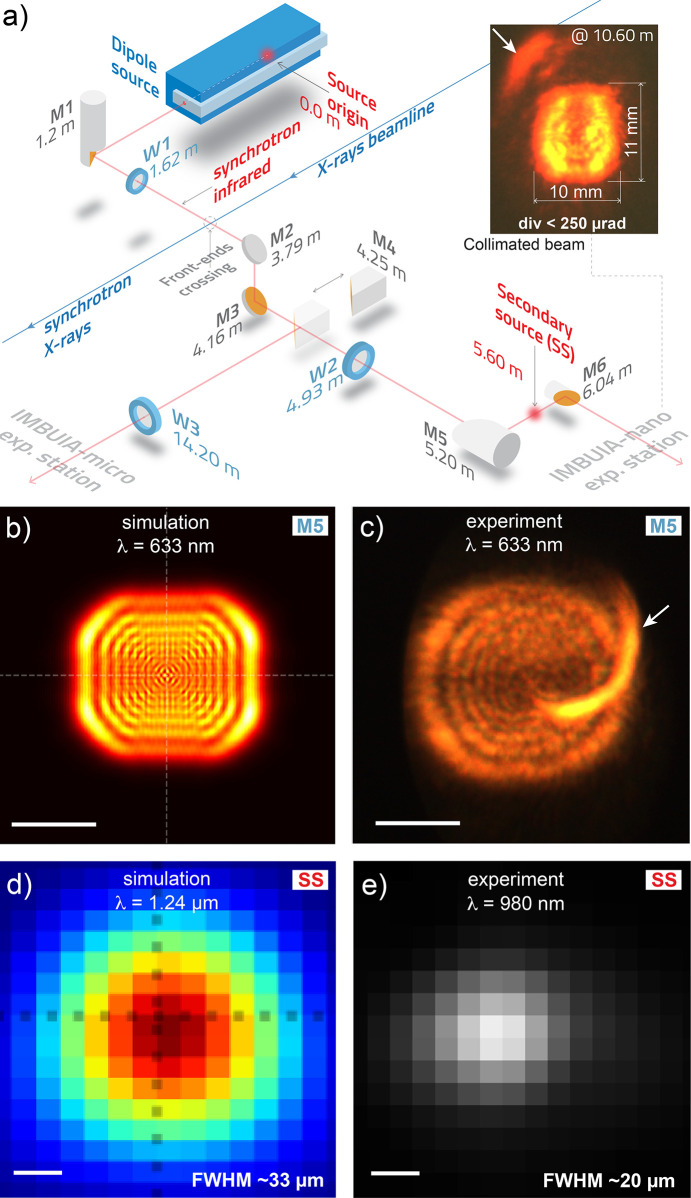
(*a*) Schematic showing the IMBUIA optical layout: three flat mirrors (M1, M2 and M3) deliver the IR broadband beam to the IMBUIA-nano station. A movable parabolic mirror (M4) collimates and directs the beam towards the IMBUIA-micro station on demand. M5 focuses the beam and M6 collimates it towards the nanoscope. (Inset) Collimated beam cross-section after M6 at ∼10 m from the source in the visible range (Si CCD camera). (*b*, *c*) Simulated and experimental beam cross-section at the M5 position for 633 nm wavelength. Scale bars represent 10 mm. (*d*, *e*) Simulation and experiment of the secondary source (SS) after the elliptical focusing mirror (M5) for 1.24 µm and 980 nm wavelengths, respectively. Scale bars represent 10 µm.

The optical layout of IMBUIA comprises four mirrors in a vacuum that deliver the broadband IR beam to either the IMBUIA-nano station or the IMBUIA-micro station [Fig. 4 ▸(*a*)]. The bending magnet IR radiation is first collected by a custom-designed water-cooled Au/Glidcop flat mirror (M1, Fig. 3 ▸), then proceeds through a 1 inch-diameter and 500 µm-thick CVD diamond window (W1) that separates the storage ring and beamline vacuum environments. Further downstream, the IR beam crosses the X-ray beam from the neighbor undulator beamline, a concept that avoids extra reflections and makes the beam path as short as possible. The beam is then directed towards a periscope made of two Au/glass 2 inch-diameter flat mirrors (M2 and M3) followed by a CaF_2_ window (W2) that separates UHV and open-air environments downstream of M3. Inside the UHV periscope, a custom-designed Au/Al parabolic mirror (M4) is mounted on a long-travel linear stage that enables beam selection to the IMBUIA-micro station, whose experiment will not be described in this work. The beam after M4 is collimated as a ∼1 inch-diameter parallel beam. Table 1 ▸ shows the full list of optical elements and their position from the source origin inside the accelerator.

On the optical table and in an air environment, a custom-designed Au/Al elliptical mirror (M5) collects the naturally divergent IR beam and focuses it 400 mm downstream, producing a secondary source (SS). Using a regular CCD and a bandpass filter centered at λ = 980 nm, we imaged the SS as shown in Fig. 4 ▸(*e*), whose shape and dimensions (FWHM ∼20 µm) are consistent with the *SRW* numerical simulation of the SS (FWHM ≃ 33 µm at λ = 1.24 µm) depicted in Fig. 4 ▸(*d*). The small discrepancy between experiment and simulation regarding the FWHM is associated with the slightly different wavelengths and exposure parameters. We used different wavelengths since we could not match available bandpass filters with the minimum energy that *SRW* can simulate. By positioning a screen at the M5 position and using a 633 nm bandpass filter, we could image the beam cross-section [Fig. 4 ▸(*c*)] of 26 mm × 23 mm, and with good agreement with the numerical prediction [Fig. 4 ▸(*b*)]. A ring structure is observed in both experiment and simulation for λ = 633 nm [Figs. 4 ▸(*b*) and 4(*c*)] and which is understood here as diffraction fringes from the bending magnet extraction port, previously presented in Fig. 1 ▸(*c*). An intense curved feature was observed [white arrow in Fig. 4 ▸(*c*)] and is interpreted as an internal reflection from the dipole chamber.

Finally, the beam is collimated by a 1 inch Au/Al parabolic mirror (M6) and then delivered to the IR nanoscope located about 2 m downstream. An image of the parallel beam cross-section is shown in the inset of Fig. 4 ▸(*a*), taken from a screen positioned ∼5 m from M6, with horizontal × vertical dimensions of 10 mm × 11 mm, and divergence <250 µrad in the visible range. Note that the parallel beam presents a similar fringes structure observed in the non-collimated beam imaged before M5 [Fig. 4 ▸(*c*)] and the curved feature (white arrow) is faded and off-centered, supporting the hypothesis of beam reflection inside the polished dipole chamber.

## 4. Experimental endstation IMBUIA-nano

**Figure 5:**
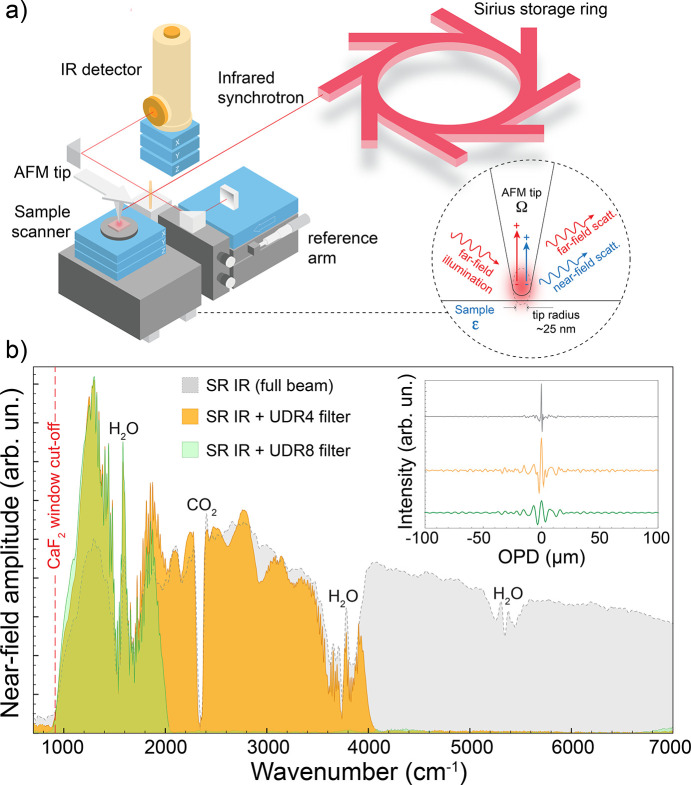
(*a*) Schematic of the synchrotron nano-FTIR experimental setup showing the broadband synchrotron IR beam that feeds an asymmetric Michelson interferometer where one of the arms is the tip-sample AFM stage. (Inset) Schematic showing how far-field broadband radiation is strongly confined at the tip apex enabling a nanometric IR probe (∼25 nm spatial resolution). (*b*) Synchrotron nano-FTIR interferograms (inset) and Fourier-processed amplitude spectra acquired from a clean gold surface. Gray, yellow and green spectra were acquired with a full broadband beam (950–10000 cm^−1^), after UDR4 bandpass filter (1000–4000 cm^−1^) and after UDR8 bandpass filter (1000–2000 cm^−1^).

The IMBUIA-nano is a station dedicated to mid-IR nano­spectroscopy in a special configuration of s-SNOM that uses synchrotron radiation as the broadband source. The collimated beam processed by the optics described in the previous section enters an asymmetric Michelson interferometer where a ZnSe beamsplitter divides the beam into a far-field movable arm (reference arm), that works as a delay line, and a static arm with an AFM stage, displayed in the schematic in Fig. 5 ▸(*a*). When illuminated by the synchrotron IR beam (far-field pink wave), the free charges of the metallic AFM tip are polarized producing an extreme field confinement at the tip apex. Therefore, the dimensions of the IR probe are no longer wavelength dependent but directly proportional to the tip radius, in this case 25 nm [Fig. 5 ▸(*a*), inset]. By bringing the sample in close proximity to the tip, an extra scattering (blue near-field scattering) is added to the overall scattering due to the near-field interaction. This last one carries local permittivity information (ɛ) from the sample surface. Far-field suppression is performed via higher-harmonics demodulation of the tip oscillation frequency, as is well established in s-SNOM.

Fig. 5 ▸(*b*) shows IR synchrotron near-field spectra taken on a gold surface, which is routinely used as a reference spectrum. The gray spectrum profile was taken using the full beam (no filters) and covers from 7000 cm^−1^ to 950 cm^−1^. The low-frequency cut-off at 950 cm^−1^ is due to the CaF_2_ window used during the commissioning period that was further replaced by a BaF_2_ window (830 cm^−1^ cut-off). The result indicates that the current optics is delivering a beam with very small chromatic aberration, as a regular tip is able to reconstruct spectra within the whole sensitivity of the mercury cadmium telluride (MCT) detector. However, the trade-off is the challenge to measure ultra-narrow interferograms [gray profile in Fig. 5 ▸(*b*), inset] due to the broadband coverage of the near-field probe. In other words, interferograms acquisition requires denser meshes of points (way beyond the Nyquist criterion) to avoid loss of information. Even operating with moderate frequency resolutions (*e.g.* 10 cm^−1^), an interferogram would need at least 4096 data points to be resolved, which is usually four times slower than standard acquisitions. Alternatively, one can tackle this challenge using mid-IR bandpass filters positioned in front of the detector, as applied in the yellow and green spectra presented in Fig. 5 ▸(*b*). In those cases, the interferograms become broader and faster to measure. Beside the acquisition time advantage, alignment is also optimized for the mid-IR range when using filters, as noticed by the improvement in the low-frequency end of the spectra. In comparison, the gray near-field amplitude spectrum is weaker than the green and the yellow spectra in the range between 1000 and 1500 cm^−1^. Therefore, the optical alignment with the full beam that produces the gray spectrum (and gray interferogram) favored higher frequencies (near-IR and visible), yielding a weaker performance in the mid-IR without filters.

## 5. Applications

It is widely acknowledged that IR analysis is a highly interdisciplinary field, and the integration of synchrotron radiation IR with s-SNOM has propelled it into the realm of super-resolved analytical tools. Moreover, with the advancement of accelerator-based technologies, which enables the production of brighter and more stable light sources, the modality of IR nano-analysis is poised for a significant leap in sensitivity. To evaluate the analytical capabilities of the IR radiation extracted from the fourth-generation synchrotron storage ring Sirius, we conducted experiments on systems comprising a polymer blend, protein nano-patches and a polar 2D crystal known to be an active nanophotonic medium in the mid-IR range.

**Figure 6:**
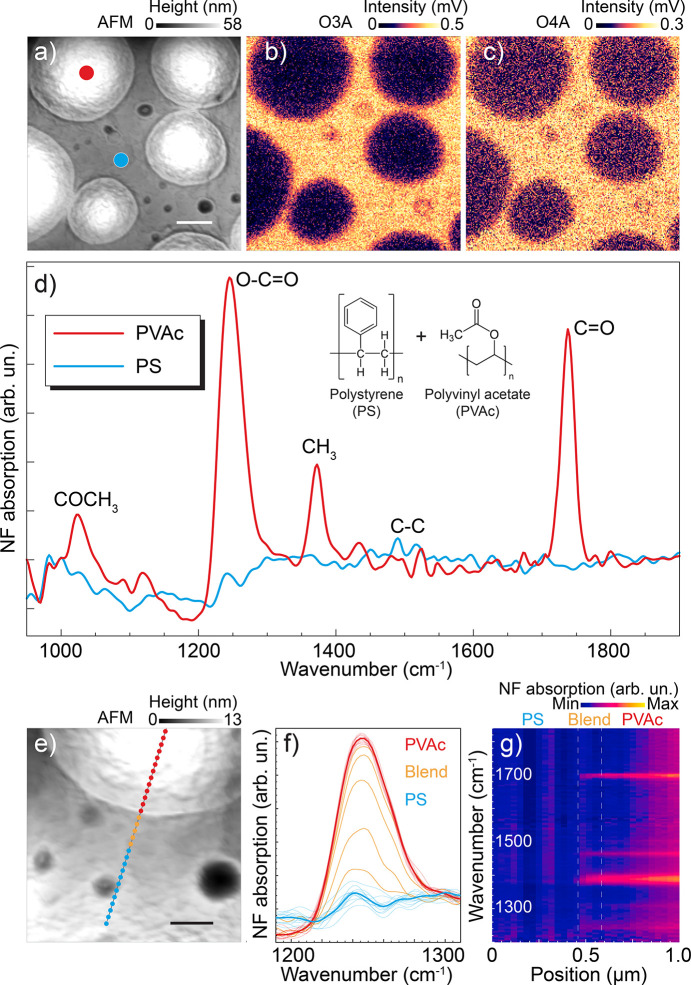
Nanoscale morphological and optical analysis of PS-PVAc films. AFM topography (*a*) and respective synchrotron radiation IR broadband reflectivity (s-SNOM amplitude) at the third (*b*) and fourth (*c*) harmonics of the tip frequency. Scale bar represents 500 nm. (*d*) Near-field (NF) absorption spectra acquired at the blue and red dots indicated in (*a*). (*e*) AFM topography revealing fine details of the film’s surface structures. Scale bar represents 200 nm. (*f*) NF absorption spectra (O—C=O stretching band) taken along the line in (*e*). (*g*) Spatial-spectral representation of the spectra taken along the line in (*e*).

Fig. 6 ▸ presents a complete topography-chemical assessment of a thin film made of a blend of polystyrene (PS) and polyvinyl acetate (PVAc). AFM topography [Fig. 6 ▸(*a*)] unveils sub-micrometre round domed structures (light gray disks) that are higher than the relatively flat surrounding PS surface. The measurement also reveals small holes in the flat part (small dark gray disks). Simultaneously to the AFM topography, broadband IR reflectivity images [Figs. 6 ▸(*b*) and 6(*c*)] were acquired in the third and fourth harmonics of the tip resonance frequency. In those images, each pixel has the whole spectrum integrated (non-interferometric). The contrast shows that the round features reflect less than the flat region, regardless of the thickness of the structures. To identify the chemical content of each topographical feature, we collected point spectra at different locations of the sample. As standard in s-SNOM analysis, both real and imaginary parts of the scattering are measured, that are related to IR reflection and absorption, respectively (Govyadinov *et al.*, 2013 ▸). Fig. 6 ▸(*d*) shows an imaginary part of the scattering point spectra (NF absorption) taken at the blue and red points indicated in Fig. 6 ▸(*a*). At the red point the spectrum presents strong absorption bands at 1250 cm^−1^ and 1730 cm^−1^, corresponding to O—C=O and C=O stretching modes. The profile also features bands at 1030 cm^−1^ and 1370 cm^−1^, which are commonly related to COCH_3_ and CH_3_ stretching modes. The set of absorption bands in the red profile are characteristic of the PVAc compound. At the blue location, only a very weak peak is noticed near 1500 cm^−1^, which can be assigned to the C—C stretching of the aromatic ring of the PS.

To further explore the nanoscale resolution of the IR probe, we zoomed into the analysis near the interface between the two phases [Fig. 6 ▸(*e*)]. A set of 30 spectra taken along a 1 µm line [red–orange–blue ≃ 33 nm step size data points in Fig. 6 ▸(*e*)] show a smooth transition between the two phases, as presented in Fig. 6 ▸(*f*), with the O—C=O absorption band monitored across the line while exiting the round structure. It is clear that the red spectra (taken within the structure) are very similar while the orange region shows a monotonic decrease of intensity as the probe is moved away (∼200 nm from the apparent topographic edge) from the structure. We assigned this behavior to a sub-surface presence of PVAc that goes beyond the topographical assessment, so it is a transition where both polymer phases coexist. Along the blue locations, the spectrum becomes constant again, but with no apparent absorption band. The same effect can be visualized for the whole spectral range in the spatio-spectral map presented in Fig. 6 ▸(*g*).

**Figure 7:**
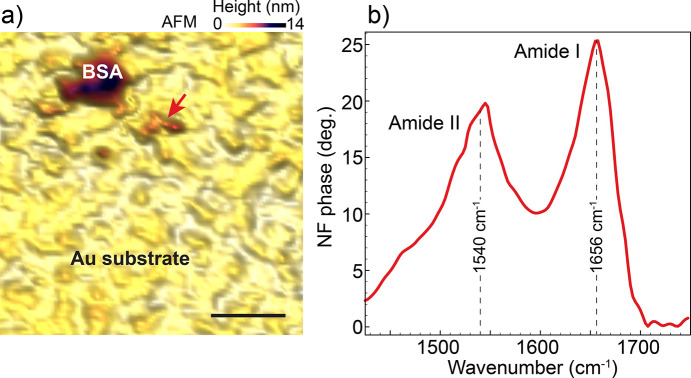
(*a*) AFM topography of BSA patches dispersed on an Au surface. Scale bar represents 300 nm. (*b*) Point nano-FTIR spectrum from the BSA structure indicated by the red arrow in (*a*).

Moving to biological systems, we analyzed the spectral response of BSA dispersed on a gold surface. In high water dilution, the dispersion of BSA produces small clusters of proteins, as visualized by AFM in Fig. 7 ▸(*a*). The sputtered gold surface has a typical roughness of ∼8 nm that, after BSA dispersion, presents patches that are commonly 5–20 nm high. Fig. 7 ▸(*b*) shows a near-field phase point spectrum taken at the red arrow indicated in Fig. 7 ▸(*a*). The spectrum features the two amide bands that are expected for pure proteins. The phase spectrum is equivalent to an absorption spectrum and the measured patch is <10 nm thick, attesting the sensitivity of the setup to small portions of biological materials.

**Figure 8:**
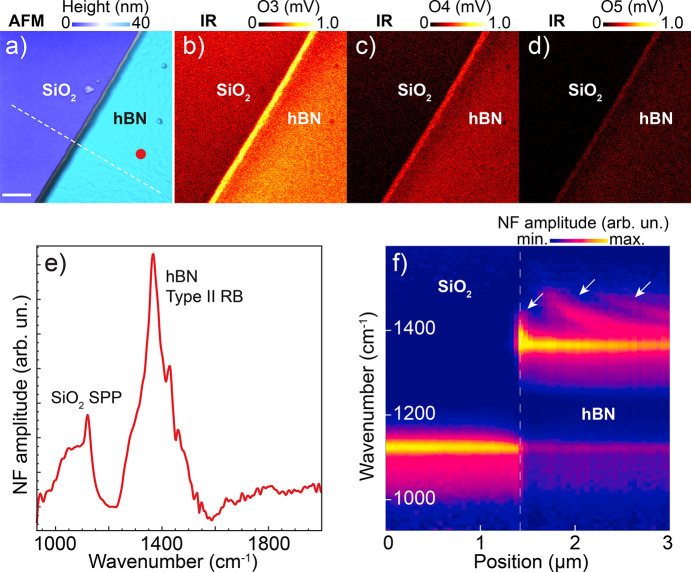
Nano-optical assessment of 2D hBN. (*a*)–(*d*) AFM topography of a ∼40 nm-thick hBN/SiO_2_ flake followed by synchrotron radiation IR broadband reflectivity maps at third, fourth and fifth tip harmonics. Scale bar represents 500 nm. (*e*) Nano-FTIR amplitude spectrum taken at the red dot in (*a*). (*f*) Spatio-spectral linescan along the white dashed line in (*a*).

Besides its sub-diffraction chemical sensitivity, s-SNOM is also well established as one of the most applied tools to study light confinement in two-dimensional materials. Its combination with synchrotron IR made feasible a series of studies in this class of materials (Barcelos *et al.*, 2020 ▸). In particular, hexagonal hBN is a strategic material for nanophotonics, as it excels at confining mid-IR phonon-polaritons in a few atoms thin crystals and can be easily integrated with other materials such as graphene. Its dielectric anisotropy enables the transport and guiding of volume waves, a key feature for future light traffic in logic circuits. Here we analyzed a 40 nm-thick hBN flake transferred onto SiO_2_ obtained by double-sided tape mechanical exfoliation, as partially AFM mapped in Fig. 8 ▸(*a*). Simultaneously to the topography measurement, broadband synchrotron radiation IR reflectivity maps were acquired at the third, fourth and fifth harmonics of the tip, as presented in Figs. 8 ▸(*b*), 8 ▸(*c*) and 8 ▸(*d*), respectively. These reflectivity maps correspond to the optical amplitude of the s-SNOM signal (O3, O4 and O5) and a contrast between hBN and SiO_2_ where the hBN is slightly more reflective when integrating the whole measured range at each pixel. A point amplitude spectrum was acquired in the middle of the hBN flake [red dot in Fig. 8 ▸(*a*)], where two peaks are observed as expected: the SiO_2_ surface phonon-polaritons (SPPs) response centered around 1100 cm^−1^ and the Reststrahlen band type II of hBN from 1250–1550 cm^−1^.

To access hBN nano-optics properties, we show a spatial-spectral scan [Fig. 8 ▸(*f*)] measured along the white dashed line in Fig. 8 ▸(*a*). The left half shows the set of spectra taken over the SiO_2_ substrate, where only the SiO_2_ SPPs were active (constant intensity below 1200 cm^−1^). Stepping on the hBN crystal, the predominant activity shifts to the range above 1300 cm^−1^, where the hBN phonon-polaritons were active. Due to the strong mode confinement and high quality factor of polaritons in hBN, the AFM tip is able to launch waves that are back-reflected by the crystal boundary. Therefore, these standing waves can be detected, as indicated by the white arrows in Fig. 8 ▸(*f*). The penetration depth of this analysis was beyond the thickness of the hBN flake (40 nm), as a faint intensity trace from SiO_2_ SPPs was still detectable from underneath the hBN.

## 6. Perspectives

Synchrotron radiation IR programs have been active since the beginning of the history of electron storage rings and have served mainly the scientific community that relies on advanced vibrational or chemical analyses. However, with the advance of accelerator-based technologies towards ultra-low-emittance machines, it is becoming more and more challenging to fit IR programs in these extremely dense magnetic lattices. This scenario is even more critical for longer waves, such as the far-IR and THz, as they require large vertical extraction ports. Consequently, new IR-THz beamlines in future fourth-generation synchrotron storage rings must be planned simultaneously with the machine conceptual design phase. In the case of the Sirius storage ring, 10% of the bending magnets were dedicated to visible-range photon diagnostics, where one of those ports feeds the IMBUIA beamline described here. Despite the outstanding performance of the current station in the mid-IR range, driven by the superior effective brilliance and stability of the source, much more can be achieved with an enlargement of the extraction port.

**Figure 9:**
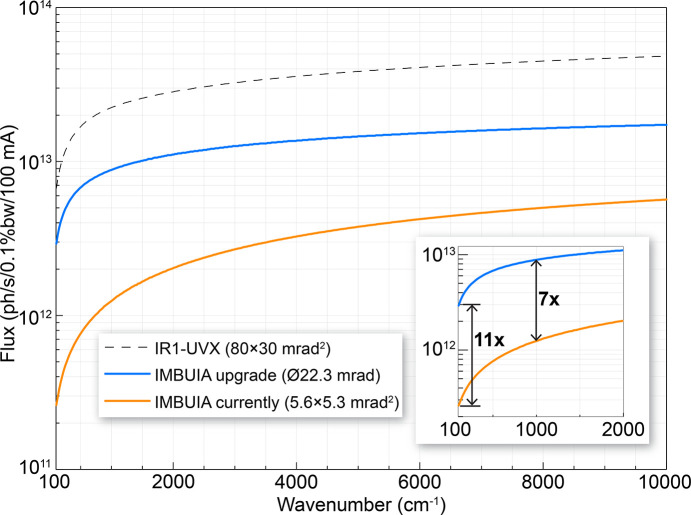
Spectral flux performance comparison between the current (orange curve), planned (blue curve) and UVX-IR1 (dashed) beamlines.

In this accelerator, the maximum gap between magnets is about 25 mm, which defines the maximum vertical aperture for radiation extraction. In Fig. 9 ▸ we present a comparison study between extracted flux from the current port of 5.6 mrad × 5.3 mrad (orange curve) and an expanded port of 22.3 mrad diameter (blue curve). Such modification would enable a flux increase of about seven times in the mid-IR range (λ = 10 µm) and about eleven times in the far-IR/THz window (λ = 100 µm), which is slightly inferior to the spectral flux delivered by the 80 mrad × 30 mrad IR port operated in our previous 1.37 GeV UVX accelerator. In a simple comparison with the current status of the beamline, the upgrade can produce a more than 300% increase in the experiment sensitivity (more than three times better signal-to-noise ratio). In the mid-IR range, the enlargement of the port would represent an increase of the numerical aperture, hence, an improvement in brilliance, which is key for advancing brilliance-demanding experiments such as s-SNOM applied to weak scatterers, in special biological systems. Yet for longer wavelengths, the upgrade will allow the extraction of far-IR/THz broadband radiation, a rather unexplored frequency range in the nano­spectroscopy modality.

## 7. Summary

In this work, we introduce an inaugural IR beamline operating within a fourth-generation synchrotron storage ring. We delve into the technical intricacies of the light source and radiation extraction, offering a comprehensive examination of the beam. The strong alignment between our measurements and simulations of the beam’s cross section at various points along the optical pathway affirms the soundness of our optical design and facilitates the coupling of the beam with the s-SNOM experiment. For our nanospectroscopy investigation, we achieved an extraordinarily wide frequency range, demonstrating exceptional optical performance in generating the focal point at the AFM tip with virtually negligible chromatic aberration across the entire MCT sensitivity spectrum. We conducted tests on diverse systems, including polymer blends, protein nano-clusters and a 2D polar crystal, yielding high-quality data. Below, we outline the key advancements realized through this work:

(i) Pioneering the first-ever IR nanospectroscopy beamline in a fourth-generation synchrotron storage ring.

(ii) Beamline radiation extraction from low-bend dipoles in a five-bend achromat lattice, an interesting option for high-energy machines.

(iii) Minimalist in-vacuum IR optics with only three flat mirrors to minimize aberrations and avoid intricate alignment procedures.

(iv) Very short optical path (crossing neighbor X-rays front-end) for achieving improved mechanical stability.

(v) Unprecedented demonstration of the brilliance power of a synchrotron, as an IR beam of only 13 µW (950–2000 cm^−1^) is capable of producing outstanding s-SNOM nanospectroscopy data.

(vi) Future enlargement of the extraction port will enable far-IR/THz nanospectroscopy with enhanced SNR.

Therefore, we foresee this work to serve as an inspiration and hope it will motivate other IR programs to evolve inside new-generation synchrotron facilities.

## Figures and Tables

**Table 1:** List of optical elements and their position from the source origin

Element	Type	Position (m)	Description
Source	Bending magnet	0.0	0.56 T B2 dipole
M1	Water-cooled flat mirror	1.20	Au/Glidcop mirror
W1	Optical window	1.62	CVD diamond
M2	Flat mirror	3.79	Au/Al mirror
M3	Flat mirror	4.16	Au/Al mirror
M4	Parabolic mirror	4.25	Au/Al mirror
W2	Optical window	4.93	CaF_2_
M5	Elliptical mirror	5.20	Au/Al mirror
SS	Focal point	5.60	Secondary source
M6	Parabolic mirror	6.04	Au/Al mirror
W3	Optical window	14.20	CaF_2_
